# Emotional dysregulation and its pathways to suicidality in a community-based sample of adolescents

**DOI:** 10.1186/s13034-023-00699-4

**Published:** 2024-01-20

**Authors:** Sabrina Mittermeier, Alexandra Seidel, Christin Scheiner, Nikolaus Kleindienst, Marcel Romanos, Arne Buerger

**Affiliations:** 1 Department of Child and Adolescent Psychiatry, Psychosomatics and Psychotherapy, Center of Mental Health, University Hospital of Wuerzburg, Margarete-Hoeppel-Platz 1, 97080 Wuerzburg, Germany; 2 German Centre of Prevention Research, University of Wuerzburg, Wuerzburg, Germany; 3 Institute of Psychosomatic Medicine and Psychotherapy, Medical Faculty Mannheim, Central Institute of Mental Health Mannheim, Heidelberg University, Heidelberg, Germany

**Keywords:** Suicidality, Emotional dysregulation, Adolescents, Nonsuicidal self-injury (NSSI), Depressiveness

## Abstract

**Objective:**

Effective suicide prevention for adolescents is urgently needed but difficult, as suicide models lack a focus on age-specific influencing factors such as emotional dysregulation. Moreover, examined predictors often do not specifically consider the contribution to the severity of suicidality.

To determine which adolescents are at high risk of more severe suicidality, we examined the association between emotional dysregulation and severity of suicidality directly as well as indirectly via depressiveness and nonsuicidal self-injury.

**Method:**

Adolescents from 18 high schools in Bavaria were included in this cross-sectional and questionnaire-based study as part of a larger prevention study. Data were collected between November 2021 and March 2022 and were analyzed from January 2023 to April 2023.

Students in the 6th or 7th grade of high school (11–14 years) were eligible to participate. A total of 2350 adolescents were surveyed and data from 2117 students were used for the analyses after excluding incomplete data sets. Our main outcome variable was severity of suicidality (*Paykel Suicide Scale, PSS*). Additionally, we assessed emotional dysregulation (*Difficulties in Emotion Regulation Scale, DERS-SF*), depressiveness (*Patient Health Questionnaire, PHQ-9*) and nonsuicidal self-injury (*Deliberate Self-Harm Inventory, DSHI*).

**Results:**

In total, 2117 adolescents (51.6% female; mean age, 12.31 years [standard deviation: 0.67]) were included in the structural equation model (SEM). Due to a clear gender-specific influence, the model was calculated separately for male and female adolescents. For male adolescents, there was a significant indirect association between emotional dysregulation and severity of suicidality, mediated by depressiveness (β = 0.15, *SE* = .03, *p* = .008). For female adolescents, there was a significant direct path from emotional dysregulation to severity of suicidality and also indirect paths via depressiveness (β = 0.12, *SE* = .05, *p* = 0.02) and NSSI (β = 0.18, *SE* = .04, *p* < .001).

**Conclusions:**

Our results suggest that gender-related risk markers in 11–14-year-olds need to be included in future suicide models to increase their predictive power. According to our findings, early detection and prevention interventions based on emotion regulation skills might be enhanced by including gender-specific adjustments for the co-occurrence of emotional dysregulation, depressiveness, and nonsuicidal self-injury in girls and the co-occurrence of emotional dysregulation and depressiveness in boys.

## Background

Suicide is the fourth leading cause of death in adolescents and young adults worldwide [1]. It is essential that suicide prevention begins before the severity of suicidality (SS) escalates from suicidal ideation (SI) to suicide attempts (SA). Therefore, we must identify which youth are at increased risk of suicidality. Previous research has identified several risk factors for suicidality [2], but given the low sensitivity and specificity of these risk factors, it is difficult to predict SA in adolescents, and the etiology of suicidality remains uncertain [3].

Suicide models such as the interpersonal-psychological theory of suicide (IPTS) [4] or the integrated motivational–volitional model of suicidal behavior (IMV) [5] focus primarily on feelings and cognitive mechanisms associated with depressiveness, including hopelessness and rumination. These models share the assumption that suicidal individuals experience very high levels of emotional distress and subsequently emotional dysregulation (ED) [6]. The specific deficits in emotion regulation (ER) processes that contribute to increased suicidality are often not integrated in the models, although several studies have demonstrated associations between aspects of ED and suicidality in adolescents [6, 7].

Longitudinal data support a causal relationship between ED and psychiatric symptomatology in adolescents [5]. Moreover, increased ED has been found to lead directly to adolescent suicidality in both clinical and nonclinical samples [6, 9, 10]. In contrast, Kranzler and colleagues found no direct association between ED and adolescent SA, but did report an indirect association via internalizing symptomatology and nonsuicidal self-injury (NSSI) [8]. Thus, the exact interplay between ED and suicidality in adolescents remains unclear. One possible mechanism underlying the relationship between ED and suicidality discussed in the literature arises in the context of the ITPS. In a sample of 151 adolescents aged between 12 and 17 years, Eaddy et al. found that the influence of ED on suicidality was explained by perceived burdensomeness and acquired capability, but not thwarted belongingness [9]. Longitudinal data in adults also support the notion that ED influences suicidality via acquired capability and the desire for suicide [10].

The role of NSSI in suicidality has been extensively studied as it is a common maladaptive coping strategy based on ED in adolescents [11], and particularly females [12]. NSSI is not only a strong predictor of the later development of suicidality [13] but also appears to contribute to the SS: In a longitudinal study of 1025 adolescents, Mars and colleagues demonstrated that NSSI significantly increased the risk of transition from SI to SA (odds ratio (OR) 2.79) [14], rendering NSSI an extremely important risk factor for suicidality. To determine which youth in this high-risk group become suicidal, it is important to understand the role of NSSI in suicidality. However, the precise mechanisms underlying the relation between NSSI, ED, and suicidality are not fully understood. Currently, NSSI is thought to mediate the relation between ED and suicidality either partially or entirely [11, 16, 17].

A further risk marker often included in suicide models is depressiveness [18–20].^1^ Indeed, major depressive disorder was found to be the most prevalent lifetime disorder in adolescents with SA [21], which may be partially attributable to the common underlying factor of ED. However, while deficits in ER can contribute to the development of depression [22], depressiveness does not appear to be a specific predictor of suicidality, as it does not differentiate between suicidal youth with vs. without SA [23, 24]. Thus, depressiveness can be seen as an important, but not unique, factor in the interaction between ED and SS.

In sum, to enable appropriate prevention strategies, detecting and predicting suicidality in adolescents is crucial, but has so far proven difficult. Previous models examined important risk factors associated with depressiveness, but only focused to a limited extent on risk markers specifically relevant to the target group of adolescents, such as ED and NSSI. The present study aims to fill this gap by using a structural equation model (SEM) to uncover the direct and indirect relationships between ED and SS in adolescents, including common mediating risk factors such as depressiveness and NSSI.

Our main hypothesis is that higher ED is directly related to higher SS. Furthermore, we expect higher ED to be related to higher levels of NSSI and depressiveness, in turn increasing the probability of higher SS by mediating the path between ED and SS.

## Methods

### Participants

The overall sample comprised n = 2350 adolescents and was drawn from the baseline survey (T0) of a cluster-randomized controlled trial evaluating the effectiveness of the universal prevention program “DUDE” [25]. The inclusion criterion was provision of informed consent from the adolescents and their legal guardians. We excluded data from individuals who missed the T0 baseline survey or did not report gender or age (n = 180). In addition, we excluded participants who were aged < 11 (n = 8) or > 14 (n = 1) years or identified as gender-diverse (n = 12). Furthermore, individuals with inconsistent responses were excluded (n = 6). Individuals with missing data in one measurement instrument were excluded from the SEM (n = 26).

The final sample comprised *n* = 2117 6th and 7th grade students from 18 different high schools in Bavaria (1092 female; 1025 male) with an age range from 11 to 14 years (mean age (*M*) = 12.31, standard deviation (*SD*) = 0.67). Subjective social status was estimated as slightly above-average (*M* = 6.31, *SD* = 1.32).

Study procedures were reviewed and approved by the Clinic for Child and Adolescent Psychiatry Wuerzburg, Germany (German Clinical Trials Register: DRKS00018945) and ethically approved by both the ethics committee in Wuerzburg (127/19-me) and the Ministry of Education and Cultural Affairs (IV.7-BO5106/200/12). Procedure-related details are available elsewhere [25].

### Measures

#### Emotional dysregulation

ED was assessed using the 18-item German adaptation of the self-report questionnaire Difficulties in Emotion Regulation Scale – Short Form (DERS-SF; Cronbach’s α = 0.91) [26], which comprises the following six subscales: (1) non-acceptance of emotional reactions, (2) difficulties in exhibiting goal-directed behavior, (3) difficulties in impulse control, (4) lack of emotional awareness, (5) limited access to ER strategies, and (6) lack of emotional clarity. Items are rated on a scale from 1 = “almost never” to 5 = “almost always”, with higher total and subscale scores indicating greater difficulties in ER. The DERS-SF is validated and widely used in adolescent studies [26, 27].

#### Severity of suicidality

The 5-item Paykel Suicide Scale (PSS) [28] assesses SS, with items increasing in intensity from SI to SA. The modified version used here contains the response options “never “, “at an earlier time point,” and “within the last 2 weeks”. For better interpretability of the model, only the data on SS in the last two weeks were considered. Although no psychometric properties can be identified in the literature [25], the PSS has been used to evaluate suicidality in both adults and adolescents [29–31].

#### Depression

The 9-item Patient Health Questionnaire measures depressive symptoms within the past two weeks (PHQ-9) [32] according to the Diagnostic and Statistical Manual of Mental Disorders, 4th Edition (DSM-IV) [33]. Items are rated from 0 = “not at all” to 3 = “almost every day”, amounting to a maximum total score of 27. For SEM, the suicidality item was excluded by subtracting it from the PHQ-9 summary score to prevent an artificially inflated covariance between depressiveness and SS. The PHQ-9 has shown good internal consistency in a school setting (Cronbach’s α = 0.84) [34].

#### Nonsuicidal self-injury

To assess the frequency of NSSI, we used a shortened 9-item adaptation of the Deliberate Self-Harm Inventory (DSHI-9) developed by Bjärehed and Lundh [35] (Cronbach’s α = 0.66—0.85). The instrument is often used in adolescents [36]. For ethical reasons, the following screening question was included at the beginning of the questionnaire: “Have you ever self-harmed, intentionally causing yourself pain? (e.g., hitting yourself, scratching yourself, burning yourself…)”. If participants responded with “no”, they were referred directly to the next questionnaire. This approach was chosen because there were concerns that presenting all self-harming behaviors could trigger some of the adolescents and thereby generate side effects, as noted by the Ministry of Education and Cultural Affairs during the study approval process.

### Data analysis

The sample size calculation can be found in the published study protocol [25].

Pearson correlations were calculated between the observed variables (point-biserial correlations for relationships with gender). To account for heterogeneity of variance, we examined gender differences regarding main model factors using Welch’s t-test.

The hypotheses were tested using a structural equation model (SEM). The measurement model for ED was based on the indicator variables corresponding to the DERS subscales. Due to content similarities, error correlations between several DERS subscales were allowed. The manifest variables depressiveness and NSSI were examined as mediators in the SEM model between ED and SS. Correlations between individual mediators were also allowed as they have long been established in the literature. The model was controlled for possible influences of age and gender, and all included variables were mean-centered.

We employed a two-stage approach, examining the measurement model first, followed by the full structural model [37]. To assess model fit, we used several indices according to current guidance on structural equation analyses [38], including the maximum-likelihood chi-square statistic (smaller and non-significant values indicate a better fit), the Comparative Fit Index (CFI) and Tucker-Lewis Index (TLI) (values ≥ 0.95 indicate good fit), the root mean square error of approximation (RMSEA) (values ≤ 0.06 indicate good fit), and the standardized root-mean-square residual (SRMR) (values ≤ 0.08 indicate good fit). Individual parameter estimates were tested using a cutoff criterion of α = 0.05. To account for zero-inflation in NSSI and SS data, we used the robust resampling method of bootstrapping with 10,000 iterations.

All statistical analyses were performed using RStudio version 4.1.2. SEM were modeled using the lavaan package (version 0.06) [39].

## Results

### Descriptive analyses and correlations

Gender-related differences in SS, ED, depressiveness, and NSSI were consistently demonstrated, with females scoring significantly higher than males (Table 1). As shown in Table 2, most correlations were significant (*r’s* ranging from 0.04—0.63). However, age was not significantly correlated with NSSI.

**Table 1:** Means and standard deviations of the main variables of the model. The results include the gender differences examined with the Welch t-test

	Male (n = 1025)	Female (n = 1092)	Welch t-test		
	Mean (Standard Deviation)	Mean (Standard Deviation)	*t*	*df*	*p*
SS	0.08 (0.45)	0.31 (0.94)	− 7.13	1596.8	< 0 .001
ED	37.28 (9.29)	41.64 (11.15)	− 9.8	2086.2	< 0.001
Depressiveness	3.9 (3.5)	5.33 (4.62)	− 8.04	2024.7	< 0.001
NSSI	0.13 (0.58)	0.25 (0.85)	− 3.74	1928.7	< 0.001

*SS*  severity of suicidality, *ED*  emotional dysregulation, *NSSI*  non-suicidal self-injury

**Table 2:** Means, standard deviations, and Pearson correlations with confidence intervals

Variable	*M*	*SD*	1	2	3	4	5
1. Gender (male/female) ^†^							
2. Age	12.31	0.67	− 0.06** [− 0.02, − 0.10]				
3. ED	39.49	10.51	0.21**[0.25, 0.17]	0.04*[0.00, 0.09]			
4. Depressiveness	4.63	4.18	0.17**[0.21, 0.13]	0.08**[0.03, 0.12]	0.63**[0.61, 0.66]		
5. NSSI	0.19	0.73	0.08**[0.12, 0.04]	0.03[− 0.02, 0.07]	0.34**[0.30, 0.38]	0.44**[0.41, 0.47]	
6. SS	0.20	0.75	0.15**[0.19, 0.11]	0.06**[0.02, 0.10]	0.35**[0.31, 0.39]	0.44**[0.40, 0.47]	0.42**[0.39, 0.46]

*M* and *SD* represent mean and standard deviation, respectively. Values in square brackets indicate the 95% confidence interval for each correlation. ^†^ indicates point-biserial correlation. * indicates *p* < 0.05. ** indicates *p* < 0.01. *ED*  emotional dysregulation, *NSSI*  nonsuicidal self-injury, *SS* severity of suicidality. Gender = males coded as 0, females coded as 1

### Structural equation model

The measurement model, consisting of the latent variable ED and including all DERS subscales as manifest variables, showed a good fit to the data (χ^*2*^ = 4313.25, *df* = 15, *p* < 0.001, CFI = 0.99, TLI = 0.99, RMSEA = 0.02, SRMR = 0.01). All standardized factor loadings of the manifest indicators were significant (*p* < 0.001), lying between β = 0.26 and 0.84.

**Fig. 1:**
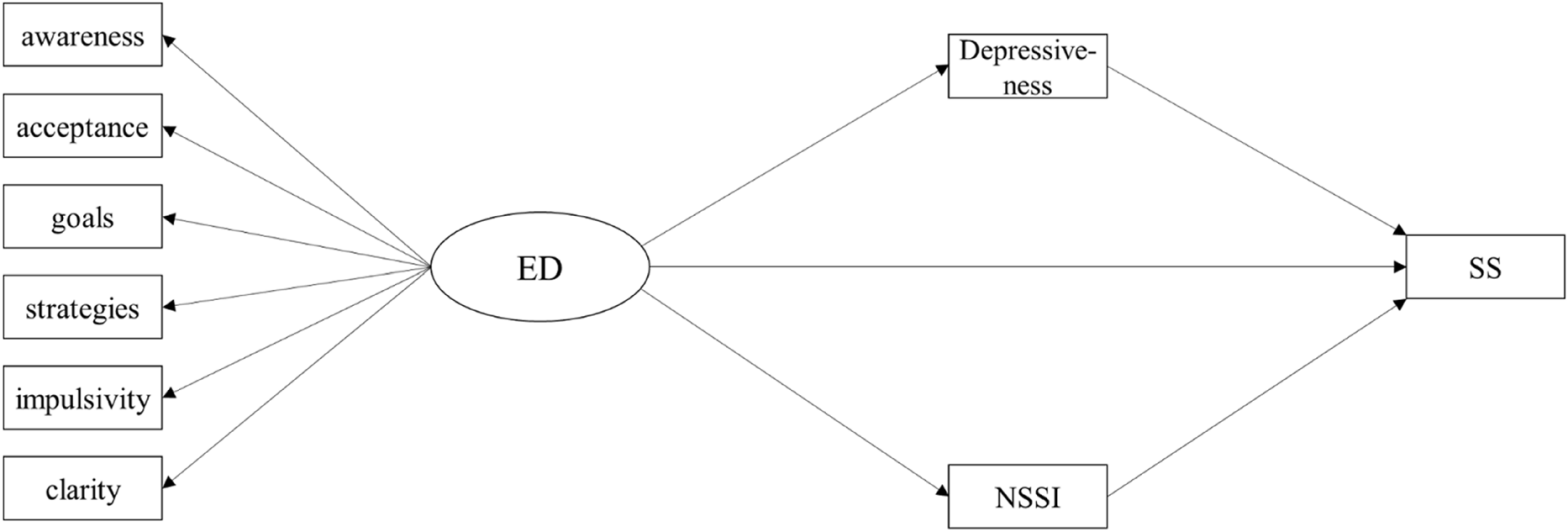
Structural equation model to examine the direct relationship between emotional dysregulation and suicidality severity and the indirect relationship via depressiveness and NSSI in youth. *ED*  emotional dysregulation, *NSSI*  nonsuicidal self-injury, *SS*  severity of suicidality, awareness = lack of emotional awareness; acceptance = non-acceptance of emotional reactions; goals = difficulties in exhibiting goal-directed behavior; strategies = limited access to emotion regulation strategies, impulsivity = difficulties in impulse control; clarity = lack of emotional clarity

**Fig. 2:**
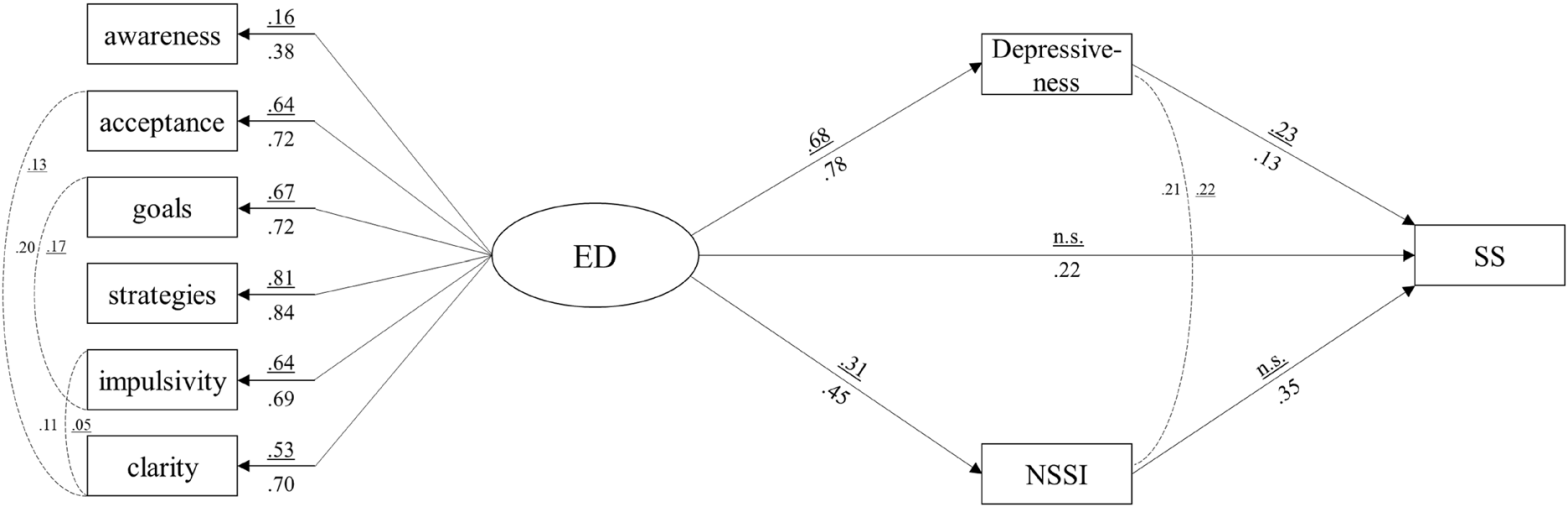
Standardized path coefficients for the structural equation model of male participants (SEMm; underlined β coefficients above the paths) and female participants (SEMf). The dashed lines represent covariances. The paths between the control variable age and the model variables are not displayed. Non-significant paths are denoted as n.s

After establishing the measurement model, we examined the hypothesized structural model (SEM, Fig. 1). When gender and age were included as covariates (SEMcov; Table 3), the bootstrapped model showed a good fit to the data (Table 3). As gender had a significant influence on the outcome variables SS (β = 0.06, *SE* = 0.03, *p* < 0.001) and ED (β = 0.21, *SE* = 0.01, *p* < 0.001), the structural model was tested separately for males (SEMm) and females (SEMf), with both gender models meeting the criteria for good fit (Table 3). All standardized factor loadings of the manifest indicators were significant (*p* < 0.001), lying between β = 0.15 and 0.81 for SEMm and between β = 0.38 and 0.83 for SEMf (Fig. 2).

**Table 3:** Model fit indices and direct, indirect and total standardized effects of model variables on SS using bootstrapping

	SEM (n = 2117)			SEMcov (n = 2117)			SEMm (n = 1025)					SEMf (n = 1092)				
Fit indices																
Chi-square (χ^*2*^), df, *p*-value	88.093	21	< 0.001***	150.77	32	< 0.001***	64.82		26		< 0.001***	76.77		26		< 0.001***
Comparative fit index (CFI)	0.99			0.98			0.98					0.99				
Tucker-Lewis index (TLI)	0.98			0.97			0.97					0.98				
Root mean square error of approximation (RMSEA)	0.04			0.04			0.04					0.04				
Standardized root mean square residual (SRMR)	0.02			0.02			0.02					0.02				
	β	*SE*	*p*	β	*SE*	*p*	β	*SE*		*p*		β	*SE*		*p*	
Direct paths																
ED → SS	0.21	0.04	< 0.001***	0.19	0.04	< 0.001***	0.11	0.04		0.096		0.22	0.07		< 0.001***	
ED → Depressiveness	0.76	0.03	< 0.001***	0.75	0.03	< 0.001***	0.68	0.03		< 0.001***		0.78	0.04		< 0.001***	
ED → NSSI	0.41	0.04	< 0.001***	0.42	0.40	< 0.001***	0.31	0.05		< 0.001***		0.45	0.06		< 0.001***	
Depressiveness → SS	0.16	0.04	< 0.001***	0.16	0.04	< 0.001***	0.23	0.06		0.006**		0.13	0.06		0.017*	
NSSI → SS	0.27	0.05	< 0.001***	0.27	0.05	< 0.001***	0.01	0.05		0.861		0.35	0.06		< 0.001***	
Indirect paths																
ED → Depressiveness → SS	0.12	0.03	< 0.001***	0.11	0.03	< 0.001***	0.15	0.03		0.007**		0.12	0.05		0.018*	
ED → NSSI → SS	0.11	0.02	< 0.001***	0.11	0.02	< 0.001***	0.00	0.01		0.867		0.18	0.04		< 0.001***	
Control variables																
Gender → SS				0.06	0.03	< 0.001***										
Gender → ED				0.21	0.01	< 0.001***										
Gender → Depressiveness				0.02	0.03	0.363										
Gender → NSSI				− 0.01	0.04	0.726										
Age → SS				0.03	0.02	0.116	-0.01	0.02		0.840		0.04	0.03		0.146	
Age → ED				0.09	0.02	< 0.001***	0.01	0.04		0.885		0.15	0.03		< 0.001***	
Age → Depressiveness				0.02	0.02	0.148	0.02	0.02		0.471		0.02	0.02		0.290	
Age → NSSI				− 0.01	0.02	0.828	-0.03	0.02		0.312		0.00	0.04		0.916	
Total effect	0.44	0.04	< 0.001***	0.39	0.04	< 0.001***	0.27	0.04		< 0.001***		0.55	0.05		< 0.001***	

^*^ indicates *p* < 0.05, ** indicates *p* < 0.01, *** indicates *p* < .001. *SEM*  hypothesized Structural Equation Model, *SEMcov*  SEM including control variables age and gender, *SEMm*  SEM based on data of male adolescents, *SEMf*  SEM based on data of female adolescents, *ED*  emotional dysregulation, *NSSI*  nonsuicidal self-injury, *SS*  severity of suicidality

For SEMm, as displayed in Fig. 2, the direct path from ED to SS was not significant after controlling for age. The bootstrapping analysis with 10,000 iterations revealed a significant indirect association between ED and SS, mediated by depressiveness (β = 0.15, *SE* = 0.03, *p* = 0.008), whereas the indirect path from ED to SS via NSSI was not significant. The whole SEMm explained 27.2% of the variance in SS.

For SEMf, we found a significant direct path from ED to SS (β = 0.22, *SE* = 0.07, *p* < 0.001) after controlling for age (β = 0.04, *SE* = 0.03, *p* = 0.15). Both indirect pathways from ED to SS—via depressiveness (β = 0.12, *SE* = 0.05, *p* = 0.02) and via NSSI (β = 0.18, *SE* = 0.04, *p* < 0.001)—were significant. The whole SEMf model explained 55.1% of the variance in SS.

## Discussion

The present study investigated which adolescents aged 11–14 years may be particularly at risk of becoming suicidal. To this aim, in a sample of 2117 adolescents, we used a SEM to examine the association between emotional dysregulation and severity of suicidality, both directly and mediated by depressiveness and NSSI.

The analysis revealed that the model should only be interpreted separately by gender: For male adolescents, we found an indirect association between ED and SS, mediated by depressiveness, whereas for female adolescents, ED was directly related to SS. Moreover, the association was further mediated by both depressiveness and NSSI in females. There were no specific ER skill deficits that especially contributed to ED and thus had a substantial impact on the model.

Our findings regarding the role of ED in relation to suicidality are consistent with previous research. A review by Turton et al. [40] showed that ED contributes to suicidality particularly in conjunction with demographic variables or other risk factors such as mental illness, but often remains non- significant as a predictor when these other factors are added to models. In our model, ED likewise only made a significant contribution to explaining the variance in suicidality in combination with other variables. On the one hand, this may be due to gender-dependent differences in ED and variables associated with the model. On the other hand, it may also be attributable to the fact that the direction of effect of ED is not clear, as ED may also play a mediating or moderating role in the system of suicidality [41]. Further research is needed to provide more precise information about the causal relationship between ED and suicidality.

When looking at the models in detail, gender must be taken into account: Gender differences in suicidality are well known [42], and research has also frequently demonstrated gender-related differences in adolescents regarding our model components ED [43], depressiveness [44], and NSSI [12]. However, these gender differences, which obviously affect suicidality, have not been previously reflected in theoretical models of suicide [45] or in most of the data-driven models beyond examining as a covariate [11, 17, 44]. Therefore, it is not possible to derive gender-specific risk markers from these models, and the individual predictive power of the models may be limited. The present study at least partially fills this gap with respect to male and female adolescents aged 11–14 years, and substantiates ED as an underlying factor for suicidality that exerts its influence via different pathways depending on gender.

Our results suggest that in girls, an elevated risk of suicidality is conferred via different pathways, i.e. not only higher ED in general, but also ED in combination with NSSI and/or depressiveness, increases the likelihood of more severe suicidality in adolescent girls. These findings correspond to previous studies with predominantly female samples, which reported individual interrelationships of the model components investigated in the present study [3]. According to our findings, all of the examined model components made an important contribution to explaining suicidality in adolescents aged 11 to 14 years. To conclusively assess the direction of effect of the model components, a longitudinal study is necessary.

In boys, the likelihood of severe suicidality appears to be increased not by ED alone but by its combination with depressiveness. It has repeatedly been documented that depression increases the likelihood of suicidality more in men (OR 6.07; 95% confidence interval (CI) 1.74–21.20) than in women (OR 4.49; 95% CI 2.18–9.23) [42]. To our knowledge, our study is the first to show that ED alone or in combination with NSSI does not increase the risk of more severe suicidality in boys aged 11–14 years, a finding that needs to be taken into account when developing suicide prevention interventions.

## Limitations

Several limitations of this study should be mentioned. First, as the data are cross-sectional, we cannot draw inferences regarding the prediction of future suicidality. Accordingly, other model constellations may also be theoretically conceived and calculated. However, to date, there is only limited literature on alternative directions of action of the model variables [10]. The analyzed model was developed on the basis of theory and existing literature, and in future research, longitudinal data need to be gathered in order to uncover causal relationships between the variables investigated. The observed variables were either assessed as trait-like symptoms with no time criterion (ED), or with reference to the last year (NSSI) or the last two weeks (depressiveness, SS), which at least suggests that there is rather one temporal direction of effect in the model.

Second, our sample encompasses an important and broad developmental period of adolescence. It cannot be assumed that the observed variables are expressed in the same way in 11-year-olds as in 14-year-olds, while at the same time, interindividually varying developmental patterns must be taken into account. We therefore calculated the model separately for each age group, and did find some differences (e.g., the covariate gender was only significant at 13 years old, and depressiveness had no significant relationship with SS in 11- and 12-year-olds). Due to the lower power and varying group sizes, we do not wish to overinterpret these differences. However, in future models, it may be useful to consider the different age groups separately, as these results provide important information for understanding the development of mental health in childhood and adolescence and might have significant implications for the development of prevention programs.

Third, the scale properties of the PSS for assessing suicidality assume a linear increase in severity, from thoughts that life is no longer worth living to suicide attempts [28], which is doubtful in clinical practice. Furthermore, the psychometric properties of the German version of the scale have not yet been sufficiently analyzed in terms of classical test theory [25]. Nevertheless, as the PSS is widely used in studies with adolescents [30, 45, 46], we retained this scale for the sake of comparability with previous research.

Finally, due to the strong influence of gender, it would have been preferable to include young people identifying as gender-diverse, but unfortunately this could not be depicted in our SEM due to the small sample size. Further research is needed in the future to examine this group more closely.

### Implications

Our results have several important implications for practice. While the vast majority of prevention programs for this age group focus on increasing knowledge and on detecting early signs of suicidality [49], our study suggests that fostering the acquisition of ER skills in adolescents may be a good starting point, as has already been successfully implemented in several programs (e.g. Youth Aware of Mental Health – YAM) [48]. The developmental phase between 11 and 17 years of age often seems to be accompanied by a decline in the use of adequate ER strategies [50], underlining the particular need for targeted age specific training in suicide prevention. Preventive measures and early detection should consider gender-specific adaptations such as the combination of ED, depressiveness, and NSSI in girls, and ED combined with depressiveness in boys.

## Conclusion

The present findings from a large community sample provide important insights into risk markers of suicidality. Our results support previous findings that ED has a substantial impact on mental health in adolescents due to its direct and/or indirect effects on the severity of suicidality. As such, our study highlights the importance of paying attention to gender-associated risk markers in a specific age group: Girls with elevated ED who show signs of depression and NSSI are at high risk of more severe suicidality and should receive appropriate prevention measures or interventions. In boys, the risk of more severe suicidality appears to be conferred primarily by the combination of ED and depressive symptoms. Disregarding gender effects in general suicide models may significantly compromise their predictive power. Future research should further examine the gender- and age-specific influence of ED on suicidality using longitudinal data across a broader age range of adolescence in order to further improve the prediction and prevention of suicidal ideation/plans and attempts.

## Data Availability

Data sharing is not possible because the data are from underage study participants and the local ethics committee has not approved the release of the data.

## Footnotes

1 ‘Depressiveness’ includes depressive symptoms that were recorded using a screening questionnaire. We refer to 'depression’ if a clinically relevant diagnosis of depression has been made [20].
